# Application of 3D Scanning as an Indirect Method to Analyze and Eliminate Errors on the Manufactured Yoke-Type Forgings Forged in SMED Device on Modernized Crank Press

**DOI:** 10.3390/ma14010137

**Published:** 2020-12-30

**Authors:** Jacek Ziemba, Marek Hawryluk, Marcin Rychlik

**Affiliations:** 1 Department of Metal Forming, Welding and Metrology, Wroclaw University of Science and Technology, 50-370 Wroclaw, Poland; marek.hawryluk@pwr.edu.pl; 2 Kuźnia Jawor S.A., 59-400 Jawor, Poland; marcinrychlik@kuznia.com.pl

**Keywords:** crank press, die forgings, joggles and shape errors, 3D scanning

## Abstract

This article proposes an indirect measurement method based on a dimensional and shape analysis of forgings for the evaluation of the manufacture and the proper operation of the key elements of the crank press, in which after modernization, a quick tool assembly based on SMED (Single Minute Exchange of Die) was implemented. As a result of the introduced changes aiming at improving the forging aggregate and increasing the production efficiency, errors were observed on the manufactured products-forgings in the form of twists and joggles. In order to solve the problem, a lot of advanced methods was used, including: dynamic system of deformation analysis, numerical modeling and as well as dimensional and shape analysis by 3d scanning. Despite the above, this approach (classic way) did not solve the problem. A proprietary method with the use of 3D reverse scanning was proposed, which allows to solve the problem of forgings errors. Based on the measurement results and analyses for a few variants of production cycles, the necessary changes were obtained, making it possible to minimize the errors and obtain proper products in respect of geometry and quality.

## 1. Introduction

Die-forging processes for large series of products use, for example, in the automotive industry are usually realized on mechanical or hydraulic presses [1]. As opposed to open-die and semi-free die forging, which makes it possible to punch large size ingots and produce “large forgings” in a small series [2], e.g., for the aircraft industry, which are produced on aggregates with high pressures and energies [3,4], such as steam-air hammers, wedge presses, etc. [5,6,7]. For that reason, die forgings obtained on presses characterize in a much larger dimensional and shape precision as well as quality compared to forgings obtained on hammers [8,9,10,11]. In the case of die forging on mechanical presses, crank presses are those usually used, mostly owing to their high efficiency with a relatively high precision of the obtained forged products [12]. However, the quality and precision of the forgings is a resultant of many factors, both technological and technical, e.g., the state and quality of the key elements of the press, such as: the slide guides, crankshafts, bearings, base holders, strands, etc. as well as the instrumentation itself [13,14]. As a result of long-term operation and wear as well as deformation of the particular components of the forging aggregate, we can observe clearances, and also macro- and microshifts of the elements cooperating in the whole system, which can result in the occurrence of errors and defects of the forgings [15,16]. A similar situation can occur also during the scheduled modernizations or other improvements of the forging machines, as well as during new start-ups e.g., as a result of insufficiently well-developed technology or improperly designed forging instrumentation [17,18].

To increase efficiency in the production process, the approach used most often is based on the ideology of SMED (Single Minute Exchange of Dies). SMED is one of the many lean production methods for reducing waste in a manufacturing process. Successful implementation of SMED requires full understanding and analysis of the retooling process as well as knowledge of the details of each operation and configuration [19,20]. According to the initiators of this issues, Shingo, a Japanese engineer [21], set up operations that were divided into two types: internal operations performed while the machine is stationary, and external operations performed during the machine is running. Wherein, in the die-forging processes, the term SMED often comes down not to the ideology, but to the nomenclature of a special system/device for quick forging tools replacement [22,23].

The implementation of such a technological solution, despite a significant acceleration of the replacement of dies, requires its adaptation to the forging unit, which in the early stage may contribute to production problems and errors of forged products [24]. In effect, this leads to obtaining less precise forgings and, in extreme cases, the occurrence of defects, such as underfills, laps, as well as twists and joggles, which partially or totally disqualify such products [25,26,27]. That is why each forging aggregate should periodically undergo a general overhaul including regeneration or replacement of the frictional elements of the lead, beside the typical current repairs. [28,29,30]. After each of such major repairs or modernization procedures of the aggregate, it is necessary to conduct a commissioning [31]. It should be added that, at present, there are no clear guidelines related to how such a commissioning should be performed. It is recommended to use the norm [32,33], which refers only to presses with low pressure forces up to 1000 tons, whereas in the case of presses with higher pressures, the acceptance conditions are different, provided by the producer of a given press, which are close to the norm: EN ISO 16092-1:2018 [34]. The whole process of the currently conducted acceptance procedures is mostly based on various measurements of the working surfaces of the table and the slide as well as errors related to the shift of those elements in respect of each other. These measurements are usually realized under static conditions, which include, beside measuring the dimensions and verifying their agreement with the figures, calibration of the control and measurement systems and verification of the key elements.

Thus far, the acceptance examinations and tests have been conducted sporadically, and they have come down to measurements of the precision and quality of manufacture as well as the parallelism of the replaced elements through their measurement and calibration of the integrated measurement systems. During the commissioning procedure, no tests related to determining the quality of the forgings produced by the aggregate are usually performed, which is mainly caused by the usually practiced variants of cold commissioning (realized at the plant of the manufacturer on a machine which does not permanently adjoin the floor, making it impossible to apply high force). Unfortunately, such standard activities do not consider the dynamic phenomena, that is an analysis determining whether the whole aggregate operates properly during forging, where the deformation lasts only 0.2 s, and high inertial forces as well as rotational masses operate, etc. [35]. This creates a situation when the aggregate works properly under static conditions, but under dynamic ones, that is during its typical operation (periodical high pressures and temperature gradients on particular elements), it can already exhibit significant deviations from the norm.

At present, owing to the progress in measurement techniques as well as other (visual) systems, it is possible to apply a whole spectrum of methods for the analysis and verification of both the elements of the forging aggregate and the apparatus as a whole [36,37]. For the measurement of the selected geometrical features crucial for the components of a forging machine, the CMM (Coordinate Measuring Machine) techniques are applied, owing to their high precision, although they are more and more often used because of the mobility as well as increasing time precision offered by the presently produced 3D scanners mounted e.g., on mobile measurement arms [38]. Of course, for calibration of the aggregate’s measuring elements, classical measurement methods are applied (rectilinearity and perpendicularity masters, gauge blocks, time sensors), as well as much more advanced systems allowing a non-contact dynamic analysis with the use of an image and structured light analysis (e.g., Aramis and others). At the stage of design, a whole range of modern methods supporting the design and analysis is applied, that is CAD (Computer-aided Design)/CAM (Computer-aided Manufacturing)/FEM (Finite Element Method) programs, which also enable an analysis of deformations and dynamic accelerations during the forging process [39,40,41,42,43]. However, often, despite the use of many modern measurement methods and advanced CMM systems, both in new installations and those after reparation, modernization or improvement, we can encounter problems resulting in geometrically and qualitatively inappropriate products of the forging process, i.e., forgings [44], whose elimination is difficult in a real process [45,46,47]. Such problems are, unfortunately, very costly for the forge, as they relate to additional shut-downs, as well as reduced production efficiency and expensive scrapping of the forgings which do not meet the quality control requirements [48].

Typically, the classical approach to solving these problems is made using the methods described above. Then, a separate dimensional and shape analysis takes place first, was made of selected elements of the press and the instrumentation with the use of different measurement methods, e.g., by use of the 3D scanning method with the purpose to analyze the geometry and construction tolerances. Then, conducting further research including an evaluation of the effect of clearances and dynamic deformations of the press as well as the force distribution during the forging process was made through numerical modeling. However, in a situation where such a typical approach using classical methods of analysis and measurement is insufficient, it is necessary to search for new non-conventional methods, which, so far, have not been applied in solving problems of this kind [49,50,51]. One of them may be the approach proposed by the authors, which consists in the use of the indirect measurement method based on measurements of selected geometrical features of the product—a yoke-type die forging, assigned for steering columns for the evaluation of the manufacture correctness of the key press elements (base holders, slide and guides) as well as the forging instrumentation mounted based on SMED.

## 2. Test Subject, Scientific Problem and Research Methodology

The main goal is presentation an indirect measurement method based on a dimensional and shape analysis of forgings (by 3D scanning), which allows to resolve problem with their twists and joggles, as a result of the modernization of the press and the implementation of the quick SMED tool change system. This proposed method enables an effective solution to the problem of forgings defects, which could not be solved in a classic way using many advanced methods by authors, including: dynamic system of deformation analysis, numerical modeling and as well as dimensional and shape analysis by 3D scanning of key elements of the press and forging equipment.

### 2.1. Determination of the Scientific Problem

**Figure 1:**
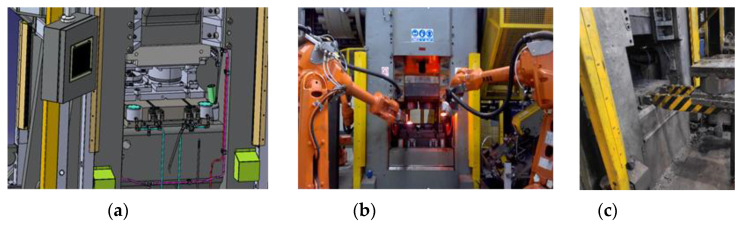
Photographs of: (**a**) the CAD model of the modernized press, (**b**) the main working portion of the modernized press during forging, (**c**) the assembly of the SMED instrumentation in the working area of the crank press.

**Figure 2:**
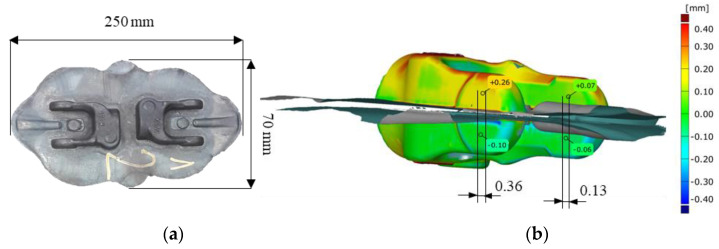
Photographs of: (**a**) a forging with the flash made on the analyzed press, (**b**) the results of scanning an exemplary defective forging with visible joggles—differences between the upper and lower portion of the forging.

The subject of investigations is a mechanical crank press Massey (Massey Forging Ltd., Hyde, England) with the load of 13MN, which underwent modernization to perform a quick replacement of instrumentation as well as robotization of the work center (Figure 1),used to manufacture a yoke-type forging in a two-component system (Figure 2a). During the start-up and testing of the modernized press, it turned out that the obtained products (forgings) exhibited defects in the form of joggles and twists, that is a shift and rotation of the upper portion of the forging in respect of the lower one in the parting plane (Figure 2b). Forgings of this type, due to their geometry and the two-component system, can cause difficulties in obtaining a proper product, that is one being within the dimensional and shape tolerance, which, for the analyzed product, equals 0.2 mm. The forgings are made of C45 steel (according to DIN 1:0503), which chemical composition was presented in Table 1.

The presented results of joggles based on scanning (Figure 2b) should make it possible to interpret the effect of the errors of the whole forging bay system (made of a press frame, a SMED system and the built-in tools) and the complex kinematic chain.

An in-depth dimensional and shape analysis of the key elements in this chain should make it possible to determine the values of corrections whose introduction into the geometry of the forging bay would correct the geometry of the produced forgings and eliminate their defects.

**Figure 3:**
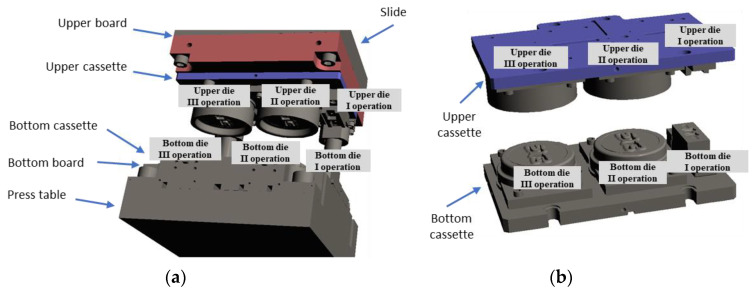
View of: (**a**) the CAD model of SMED system installed in main boards, (**b**) the CAD model of SMED system with the mounted tools assigned for forging in three operations.

Figure 3b shows an SMED system with the mounted tools assigned for forging in three operations. This instrument is built inside the work center in such a way that the upper board is permanently connected to the slide by means of a stake keying (Figure 3a), which is responsible for their positioning in respect of each other. The upper case is mounted to the upper board through a keyways/cleft joints and screws, connected to the upper tools. In turn, the lower board of the case, to which the lower tools are fixed, is mounted to the lower board also by means of a cleft joint: after the case has been driven into the key seats on feed rolls and reached the end stops, where it is blocked by the side wedges. The lower board is connected to the press table in a similar way to the upper one.

To increase the precision of movement, the upper and lower board of the SMED system itself are joined by means of two massive poles visible in Figure 3a. For a press constructed this way, together with the SMED system and the described joints in the whole kinematic chain, errors occurred on the forgings in the form of joggles or joggles with twists (Figure 2b). Unfortunately, defects of this type are very difficult to eliminate and they are formed because of a combination of factors and errors resulting from the manufacture tolerance of the component elements of the modernized press. The first error component is related with the press itself. The geometry of the slides in the press undergoes wear during the operation of the given tool. The occurring clearances are cancelled through regulation of the wear limit (by means of patterns, e.g., feeler gauges, gauge blocks). After the guides have crossed the minimal dimension of thickness or flatness, they undergo a general overhaul.

However, this prevents the slide shifting along the guides from moving with an ideal rectilinear trajectory. The press, after each repair, is commissioned according to the norms, which pay a special attention to the measurement of the geometrical dimension components that describe the movement errors of the slide in respect of the press table, which enables an objective commissioning of the press. The second error component in the analyzed solution refers to the connection with the replaceable SMED system with the permanently mounted boards: the slide and the press table. By assumption, the geometry of these boards, as well as the SMED tool itself, are made according to the technical documentation, which assumes adequate manufacture tolerances for individual elements and dimensional chains. The individual errors of the controlled geometrical features of the elements are within the assumed tolerances. The third error component results from the precision of the forging instrumentation manufacture. An additional factor affecting the formation of the joggle error is the fact that the forging process is a dynamic phenomenon. The occurring forces cause elastic deformations of the individual elements of the instrumentation, whose presence is difficult to determine and consider at the stage of design, or during static measurements. Also, the occurrence of high side (horizontal) forces during the making of a forging in a two-component system should be taken into consideration, because the forging is a long and thin element (Figure 2a).

### 2.2. Scientific Assumptions and Methods

The determination of the causes of error resulting in the production of defective forgings was conducted in several stages. At the first stage, a standard (norm-based) measurement of the key elements of the press was made, including the slide, the table and the guides, with the use of classic measurement methods, as well as the component elements of SMED and the whole system in an open and close position, after the assembly on the press by means of 3D scanning. The following stage involved an analysis supported by numerical modeling of the process, as well as with the use of a fast system of measuring the dynamic deformations. The last key stage was, however, the application of the indirect method proposed by the authors, consisting in a measurement of the forging defects—joggles of the forgings made on the press, which provided a possibility to propose a solution of eliminating the geometrical errors of the products.

What is more, the possibility of occurring joggles of the slide and table axis as a result of elastic deformations in the mechanisms of the press was excluded due to the frame characterizing in high rigidness. It was assumed that in the case of insufficient rigidness of the frame, a problem with the obtained precision would have been encountered before the modernization of the station.

For the measurements of the forgings collected from the forging process and the component elements of the forging aggregate, a measuring arm ROMER Absolute ARM 7520si (Hexagon Manufacturing Intelligence, Switzerland) with an integrated RS3 scanner as well as the Polyworks 2015 software enabling 3D scanning were selected. The device makes it possible to perform contact measurements with the use of a contact measuring probe as well as noncontact measurements by means of a linear laser scanner RS3 integrated with the arm, which provides the possibility to collect up to 460,000 points/s for 4600 points on the line with the linear frequency of 100 Hz and with the declared measurement accuracy of the scanning system agreeing with the norm B89.4.22, equaling 0.053 mm.

For the numerical modelling of the forging process, the computing package ForgeNxt3.0 was used. To compare some of modelling, Qform VX 9.0.10.1 was used with conditions similar to Forge Nxt 3.0 software. The following boundary-initial conditions were assumed. A thermomechanical 3D model with rigid tools (die inserts—elements with a heat exchange) was developed. The geometry of the tools—the preform as well as the other technological parameters of the process—were implemented into the program based on the original 3D models and operation sheets. For the discretization of all the tools, 488,346 elements of the QUAD4 type were applied, whereas for the charge—16,532 elements, also of the QUAD4 type. For input material, automatic grid remeshing and resoning was applied. The shift velocity of the upper die inserts was assumed according to the kinematic parameters of the applied crank press Massey 13MN (Massey Forging Ltd., Hyde, England). The tribological conditions were assumed based on the Tresca bilinear friction model with the factor of 0.3 for all the work surfaces of the tools, which was identical like in the industrial process, where a lubricant of a water with graphite solution was applied. The temperatures of the tools were assumed, measured by means of a thermovision camera Flir T440, (FLIR Systems, Inc. Wilsonville, OR, USA) equaled 250 °C, and of the charge material—1150 °C. The times of the consecutive operations were determined by means of a camera with the option of recording 300 frames per second. The recorded average forging cycle of one forging (three operations) at automatic work equaled 17 s. The heat transfer coefficients in the contact between the charge material and the tool material, as well as with the environment were assumed as follows: 25 and 0.4 kW/m^2^K, respectively. The Forge software uses the Norton–Hoff law of plastic flow as a constitutive equation. The model of the material, steel C45, was collected from the Forge “FPD Base 1.3” database. Detailed information related to additional conditions can be found in [52].

For the precision analysis of the press movement together with the mounted forging instrumentation, the mobile system ARAMIS (GOM mbH, Braunscweig, Germany) was used for non-contact three-dimensional dynamic measurements, in the variant equipped with video cameras with the resolution of 6 Mpx and a data acquisition module GOM Testing Controller v6.3.0, which makes it possible to take photographs with the maximum resolution of 335 Hz. In the applied configuration, the system provides the possibility to perform measurements in the measurement field of 1110 × 1110 × 1345 mm. The system makes it possible to perform dynamic measurements of objects with complex geometry and large sizes. Based on the photographs taken by means of digital cameras, the system recognizes the surface structure of the measured object or the special markers (relevant coordinates are assigned to each pixel in the photo). The system, after a sequence of movement, analyzes the recorded image and calculates the displacement and deformations on their basis [53].

## 3. Discussion of Results

In the first place, the agreement of the manufacture of the particular component elements of the SMED device was analyzed separately, based on the technical documentation, as well as in the open and closed position on the press with the use of 3D scanning. Next an evaluation of the effect of clearances and dynamic deformations of the press as well as the force distribution, displacements, velocity of material flows during the forging process was made through FE modelling. Finally, due to the lack of satisfactory results, the proprietary method of indirect measurement with the use of 3D reverse scanning was proposed, which allowed to solve the problem of errors of forgings and, on this basis, to introduce improvements and design changes.

### 3.1. Results of Analyses for Particular CMED System Components

**Figure 4:**
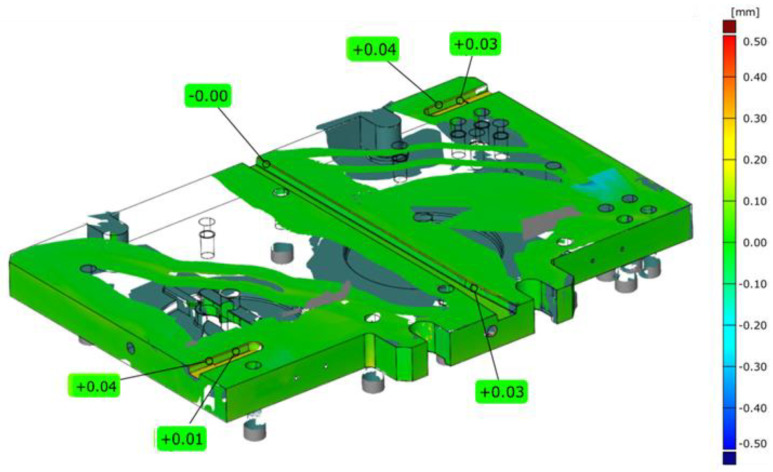
An example of measurements with view of colour deviation map of 3D scans of lower cassette.

To verify the press according to the norm ISO 6899:1984, examinations of the perpendicularity of the upper board in respect of the lower one were performed through a measurement by means of classical metrological tools. A time sensor was permanently fixed to the upper surface in the central point of the press, and an angular gauge was placed on the lower one. Through the movement of the slide, the deviations on the sensor were verified in the whole stroke range of the device. The measured perpendicularity deviations on the length of 254 mm did not exceed the value of 0.03 mm, either in the right-sided or left-sided measurement. Additionally, with the use of the 3D scanning method, a dimensional control of the key elements being a part of the SME system was performed. Figure 4 shows exemplary deviation maps (SMED system components-) of the case and the upper board presented in reference to the nominal CAD model.

The analyses of the performed measurements made it possible to clearly confirm that these elements had been manufactured according to the technological documentation. The selected key geometrical features were within the assumed fields of dimensional and shape tolerances. For example, the most important width of the key groove measured by way of scanning equaled: 0.05 mm, respectively, in reference to the figure tolerances equaling: 0.08 mm. The deviations from the nominal CAD model visible in the figures in the form of the blue and red colour prove the presence of acceptable deviations on the surfaces (non-cooperating), for which only roughing was to be applied. However, it should be noted that such a geometrical analysis of the components, even deepened by an analysis of the dimensional chains, does not make it possible to obtain a clear answer to whether the horizontal side forces as well as the inertial forces occurring during the forging process can negatively affect the obtained geometry of the product. It should also be emphasized that the quick exchange system SMED was designed as a prototype and this can also provide results which are difficult to analyze. That is why the next stage of research was performing 3D scanning of the SMED device mounted on the press.

### 3.2. Analysis Results for Particular SMED System Components in the Press, in the Upper and Lower Slide Position

**Figure 5:**
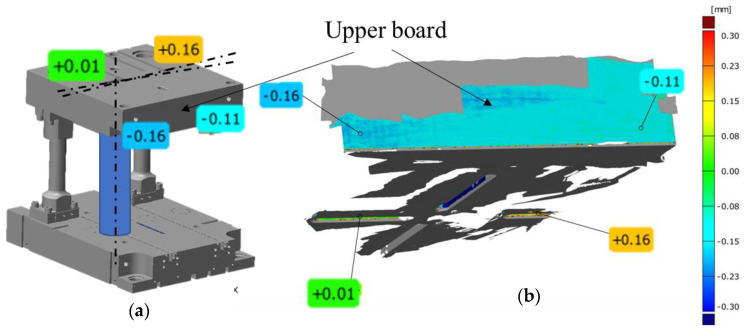
Results of the analysis of the change in the position of the SMED system components on the press in the simulated forging process: (**a**) diagram, (**b**) color map of deviations with 3D scanning results.

The following step in the measurements of the analyzed forging bay was an analysis of the position change of the SMED system components on the press in the simulated forging process, because the guides in the press have the largest clearance in its upper portion and this value decreases with the slide’s movement downwards, towards the contact with the tools. The differences after the slide comes back to the top upper position can result from a different position which it assumes then, where the initial movement has no significant effect on the process. It is, however, crucial that the system move repeatedly in the lower extreme position of the slide. To that end, the 3D scanning method was applied to analyze the position in the lower extreme. In this case, there is no possibility to perform such a measurement in the real forging process. To simulate the forging process, a decision was made to cancel the clearances on a special element shown in Figure 5a, placed in the area where the finishing tools should be located. A series of measurements was made for the same load to determine the repeatability. The performed measurements made it possible to obtain a colour map of deviations (Figure 5b).

Based on the representative results, we can notice that, after the housing of the SMED device has been closed (without the tool cases), a shift from the value of 0.11 mm to 0.16 mm takes place to the right, causing a twist by a small angle value visible on the front of the board. However, while analyzing the position of one of the side walls of the cotter beds on the upper SMED board (view from the bottom), we can notice a shift from the nominal CAD values to the values from +0.06 mm to 0.16 mm, which makes it possible to mark the potential position of the axis of the board’s twist. The performed analysis for a series of measurements confirms that the slide together with the SMED upper board moves repeatedly in the forging process.

### 3.3. Results of Dynamic Displacements

**Figure 6:**
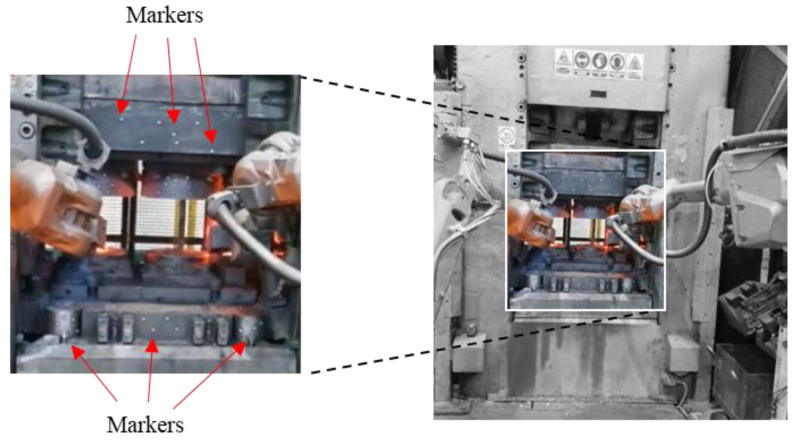
View of the press frame with visible markers.

Independently of the obtained 3D scanning results, an attempt was made to analyze the displacement of the so-called “marker—reflective—points”—placed on different elements of the press to examine the latter’s rigidity as well as the distribution of forces during the forging process. Figure 6 shows the measurement station used to examine the movement dynamics of the robotized press during forging as well as a diagram of the cases with tools with superimposed reference points. The environment in which the measurements are performed is burdened with a large amount of vibrations, which causes movement of the base in respect of the sensor of the Aramis (GOM mbH, Braunscweig, Germany) measurement system. To eliminate them, measurements were carried out with the use of an algorithm of movement correction of the rigid body incorporated into the measuring device based on the measurement of the reference points connected with the base.

**Figure 7:**
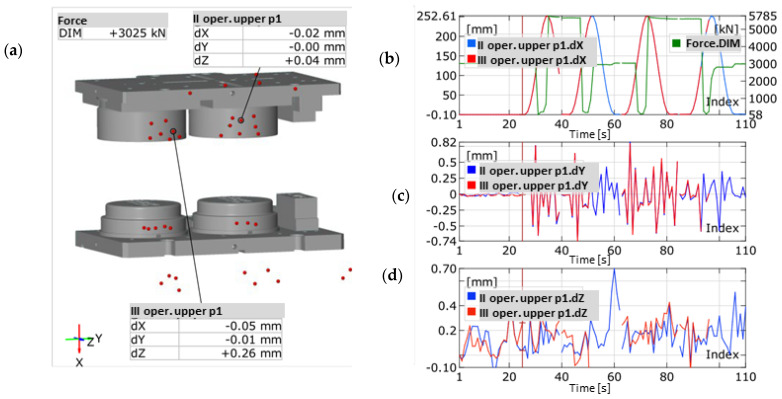
Example of the use of dynamic systems for the measurements of the forces and displacements in a forging operation: (**a**) measurement result for an open press, (**b**) change in the position value in axis X of selected reference points, (**c**) change in the position value in axis Y of selected reference points, (**d**) change in the position value in axis Z of selected reference points.

In order to perform an analysis during the forging process, a measurement of four forging cycles was made, visible in the form of sinusoidal changes in the values of component position x of the selected reference points connected with the upper section (Figure 7b).

The performed measurements made it possible to record three components of displacement of the reference points connected with the tools mounted in the cases (Figure 7b,c). The maximal force values were implemented through communication with the machine equipped with tensometric sensors and correlated with the analysis through the element connecting the systems, which recalculates the crank angle into the movement of the slide. Figure 7 also shows the assembly of the upper and lower case, where we can see the shift component values at a given moment of the forging press slide movement. The press body was assumed as an immobile base and the shifts of the upper portion were examined. The press stroke was assumed to be about 252 mm, which is in accordance with the machine specification, where the nominal value is 254 mm, with an option of regulation +/−6.5 mm, depending on the height of the forging tools. The maximal deviations in direction Y, being transverse to the machine operator, oscillated within the scope of up to 0.8 mm (Figure 7b). The result occurred in the area of free movement, where the guidance was exclusively the effect of the cooperation between the slide guides and the upper and lower board columns. At the moment of contact between the mountings guiding the tools, before the contact with the material, the clearance was minimized to 0.25 mm, with the tendency for shifts towards the finishing impression at the final stage of forging.

In the direction of axis Z (Figure 7d), being longitudinal in respect of the operator, the indications equaled 0.4 mm in the direction of the operator in a dead motion and 0.1 mm in contact as well as at the end of the process. The displacements after a few beats of the press, showing translation in the extreme open position gave the following results: 0.00 mm in Y and 0.04 mm in Z for the rouging insert as well as 0.01 mm in Y and 0.26 mm in Z for the finishing insert, in the case of a static measurement (Figure 7c,d). This confirms that the guides in the press have different clearances, depending on the height. The clearance is the smallest at the height of the slide’s movement for the close press.

**Figure 8:**
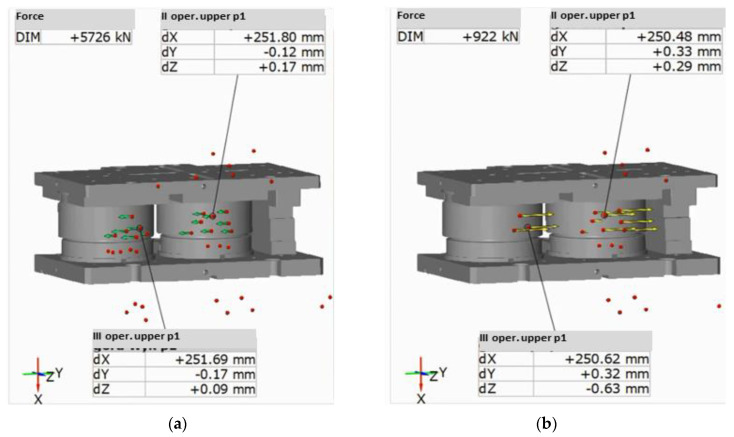
Vectors of upper insert displacements: (**a**) before reaching the lower stroke position (dx 251.69), (**b**) after reaching the lower stroke position (dx 250.62).

The maximal deflection in 60 s of movement in the direction of Z probably resulted from the resonance caused by the machines at a small distance from the examined area. Additionally, we see that the recorded diagrams are characterized in significant randomness, which makes a thorough analysis difficult. An analysis of the shift values at the moment of contact between the tools and the formed material as well as directly after its end was performed (Figure 8). The measurement method was based on dividing the examined points into the upper and lower ones, where the lower parts were assumed as an immobile base, and the upper parts—as free ones.

At the moment of reaching the extreme lower point of the press slide, the upper tools demonstrated deflections of 0.12 mm on the roughing tool and 0.17 mm on the finishing tool, in the direction of the left side of the press, as well as 0.17 mm and 0.09 mm in the direction of the operator (Figure 8). After the end of the cycle and the beginning of the return movement, the values increased, with a change in the direction to 0.33 mm and 0.32 mm towards the right side of the press, and 0.29 mm towards the operator for the second operation, while 0.63 mm for the third one.

**Figure 9:**
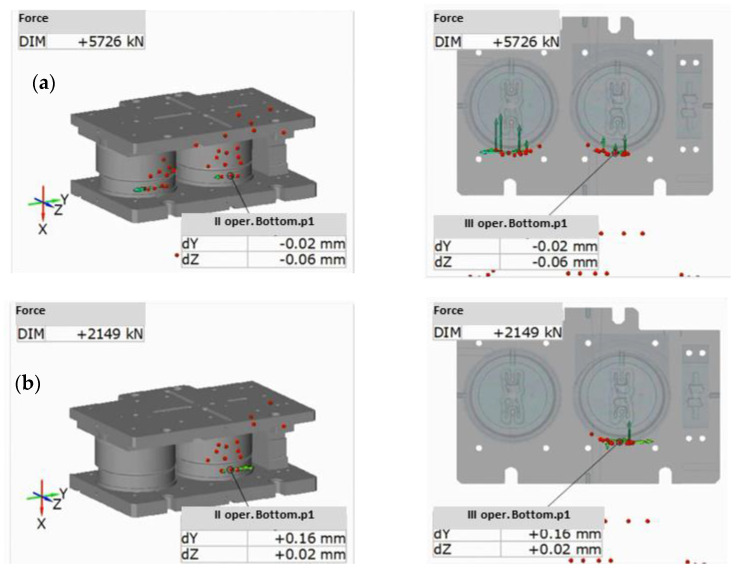
Vectors of lower insert displacements: (**a**) before reaching the lower stroke position, (**b**) after reaching the lower stroke position.

Much smaller deflections were demonstrated by the examination of the second part, where the same points on press body were assumed as the base, while the displacements of the lower section underwent verification (Figure 9). The values were verified at the moment of active forming on the roughing insert demonstrating the largest deviations. The displacement towards the back of the press equaled 0.02 mm towards the left side of the device before forming (Figure 9a) and 0.16 towards the right side after forging during the return cycle (Figure 9b). In the longitudinal directions, the values were: 0.06 towards the back of the press and 0.02 towards the operator. A comparison of both variants shows a much larger rigidity of the lower tool packets as well as a tendency to maintain the base position. This is caused mainly by the static assembly of this portion as opposed to the upper ones, where the dynamics cause larger deviations from the base axis.

**Figure 10:**
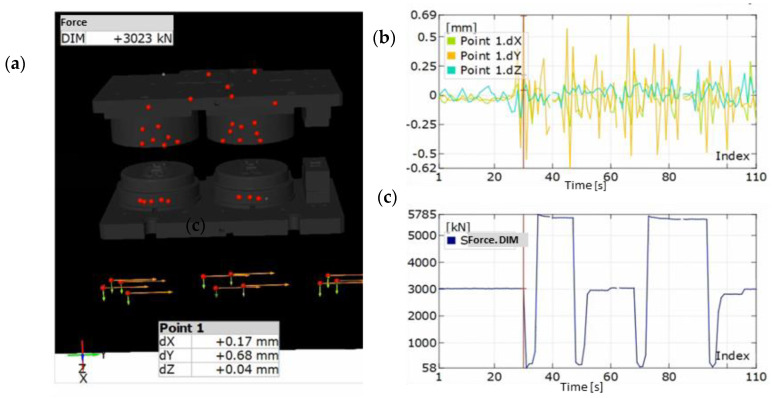
The view of: (**a**) analysis of vibrations of the measuring device without a load, (**b**) change in the position value in axis X, Y and Z, (**c**) change of force value in the process.

The examinations included an analysis of the vibrations of the base and the measurement results were corrected by means of an algorithm for the movement correction of the rigid body built into the measuring device (Figure 10). The obtained results point to a large measurement error in the process without a load, where the vibrating masses as well as the machines located in the environment (double-acting stamps and crank presses) introduced significant vibrations onto the floor on which the measurement was being made. In the extreme point of movement, the effect of vibrations on the position of the scanner equaled up to 0.7 mm in respect of the base position, from which the image calibration was performed.

In view of the measurement results correlated with the variables introduced through the floor’s operation onto the measurement device, the implemented method should not be a basis for the determination of the displacements, as the obtained results can significantly deviate from reality, depending on the floor’s vibration level at the moment of measurement. In turn, in the case of a prolonged examination, owing to which it is possible to obtain the exact course of vibrations, and assuming a harmonic and repeatable operation of the machines around the examined area, it is theoretically possible to perform an analysis by introducing a correction of movement based on the measured values.

However, it is difficult to determine whether the introduced corrections enable a complete elimination of the floor vibrations and how much this affects the measurement results. In the examined case, due to the character of randomness, the reading error of indications was not eliminated.

### 3.4. Numerical Modelling Results

**Figure 11:**
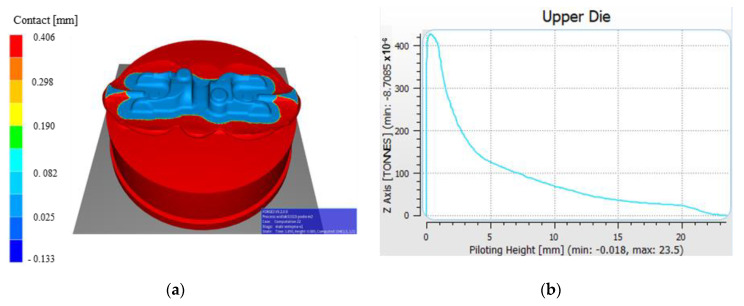
Results of analysis with the use of numerical modelling: (**a**) contact of the forging after the second forging operation, (**b**) course of forging forces in the function of displacement in the vertical direction.

Numerical modelling of the forging process was also performed in order to determine the value of horizontal forces occurring during forging, especially during the roughing operation, where the highest vertical forces were recorded (along axis Z), which equalled over 400 tons (Figure 11). Even though the side (horizontal) force values are not high in respect of the vertical forces, considering the dynamic character of the forging process (deformation time 0.2 s), they can be of significant importance, especially in the case of small clearances between the particular elements of the press itself, as well as the connection of the press with the SMED device.

**Figure 12:**
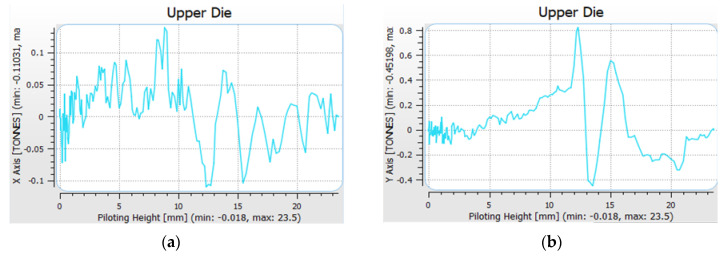
Results of analysis with the use of numerical modelling: (**a**) course of forging forces in the function of displacement in the horizontal direction in axis X, (**b**) in the horizontal direction in axis Y.

In turn, the values of horizontal forces were much lower: 0.8 ton (along axis X) and a little over 1 ton (along axis Y), which has been shown in Figure 12.

**Figure 13:**
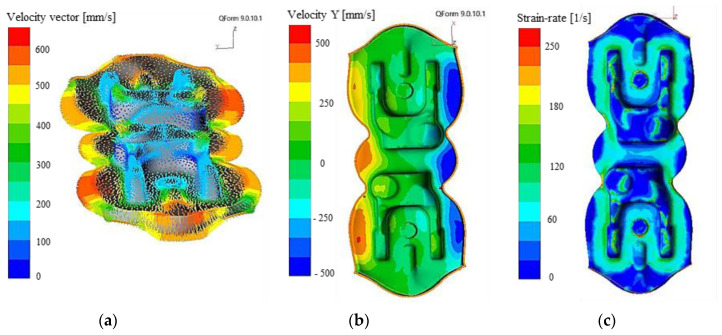
Results of numerical modelling: (**a**) material flow rate during roughing, (**b**) material flow rate in the transverse direction, (**c**) deformation rate.

Also, the vectors of the material’s displacement rates in the roughing operation were analyzed, where the material deformation is the most significant. The analysis showed a high coefficient oscillating around the values of about 500 mm/s near the forging’s arms, with a tendency for clockwise twisting (Figure 13a). After the analysis of the flow rate between the sides of the forging (Figure 13a), we can observe a difference at the level of 50–80 mm/s, which implies a tendency for unidirectional elastic rotation of the upper tools in respect of the lower ones in the production cycle. This is confirmed by the analysis of the rate distribution in direction Y (Figure 13b), as based on the results, we can notice that the highest rate values (with opposite signs) can be observed in the lower left and upper right portion. In addition, the high deformation rate in the roughing operation in some areas equal almost 200 s^−1^ points to high deformation rates, which can be the cause of defects on the forgings in combination with big masses and inertia.

**Figure 14:**
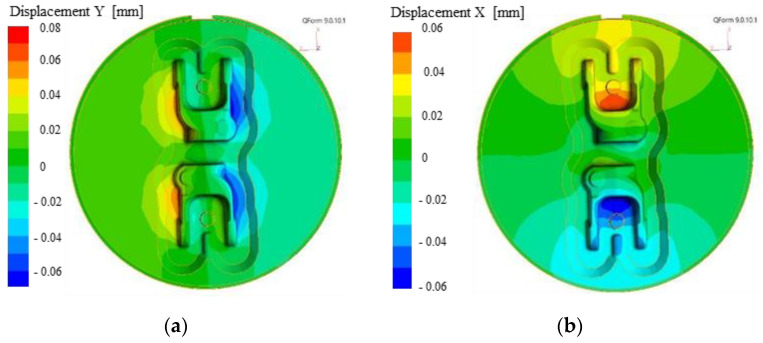
Results of modelling: (**a**) transverse displacement of the tool, (**b**) longitudinal displacement of the tool.

To confirm the results obtained on the forging model, a simulation was also performed on the tools by means of an analysis of deformations caused by the forging force (Figure 14). Only the results of the selected directions of horizontal displacements have been intentionally analyzed to present the quantities influencing the analyzed issue. Considering the data in the XYZ system is complicated, and displacements in the Z direction have no significant effect on the horizontal displacements. The results obtained in the tool analysis process confirm the results obtained on the analyzed forgings.

In the case of the transverse direction, the values were estimated at the level of 0.07 mm in the direction which is in accordance with the rate results on the forging, and 0.04 in the opposite directions. As such, a larger component of the force is applied from one side, which causes lack of symmetry of the system and can result in a tendency for twisting in the dynamic movement. For the other examine direction, the displacements were maintained at the level of 0.05 mm on both sides with a slight angular shift of the central point in the direction identical to the transverse shifts.

Based on the presented test and simulation results, it can be stated that the classic approach to solving such a complicated issue, due to very complex kinematic chains, is not sufficient. The performed measurements with the use of 3D scanning techniques of the particular key elements of the system which can possibly cause forging defects did not show any differences in respect of the technical documentation, and their values are within the assumed tolerance fields. It should be noted that the observed values of joggles and twists are relatively small (at the level of 0.2–0.3 mm) in comparison to the sizes of the key elements of the press and instrumentations, where the manufacture tolerance for the smallest elements from the kinematic chain (base holders) is at half of this level. In turn, in the case of larger table tops, the manufacture tolerance is at the level of 0.05–0.2 mm. Nevertheless, as it was demonstrated by examinations with the use of numerical modelling of the forging process, as well as a system for dynamic measurements of displacements, in the case of a long forging of this type, horizontal side forces occur, which can be of importance in terms of high deformation rates (0.2 s) and very high inertia of the whole system.

To analyze the errors in the position of the boards facilitating the tool replacement (from the SMED system) in respect of each other, a decision was made to use the forgings. This method will make it possible to clearly determine the shift of the die set during the forging process, which causes (direct effect on the element quality) the specific errors in the form of joggles visible on the forgings. An advantage of the possibility to measure only the forgings is the fact that their dimensions are relatively small and can be collected without difficulties from the forging process. These forgings describe the state of the forging aggregate in the extreme lower position of the slide under load. To eliminate random errors, an analysis will be performed on more series of periodically collected forgings and the measurement values will be averaged. Scanning the collected forgings will make it possible to determine the dimensions of the joggles, whose analysis will enable a correction of geometry, which, introduced into the forging bay, will also make it possible to obtain a forging being within the required dimensional tolerance scope.

### 3.5. Application of the Indirect Method Based on 3D Scanning of a Forging Set

**Figure 15:**
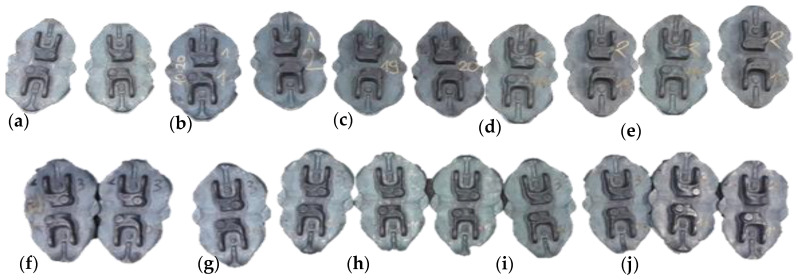
View of two consecutive forgings collected from the process: (**a**) the 100th and 101st item from series I, (**b**) the 200th and 201st item from series I, (**c**) the 300th and 301st item from series I, (**d**) the 400th and 401st item from series I, (**e**) the 500th and 501st item from series I, (**f**) the 100th and 101st item from series II, (**g**) the 200th and 201st item from series II, (**h**) the 300th and 301st item from series II, (**i**) the 400th and 401st item from series II, (**j**) the 500th and 501st item from series II.

Figure 15 shows photographs of two series of sets of cyclically collected pairs of consecutive forgings for the analysis of the joggle parameter with the use of 3D scanning. Both in the case of the first and the second series, every 100 items, two consecutive forgings were collected, to eliminate random errors (for example, the 100th and the 101th forging were collected), up to 500 items from the process, from instrumentation with geometrical joggle defects (dimensions before the correction—series I). The size of each series was assumed to 500 items. After that period, based on the analysis, intensive wear of the tools occurs, which could interfere with the analyses.

The second series (series II) of two consecutive forgings (Figure 15f–j) was collected in a similar cyclic manner after introducing a correction of the instrumentation geometry to verify the properness of the introduced changes.

**Figure 16:**
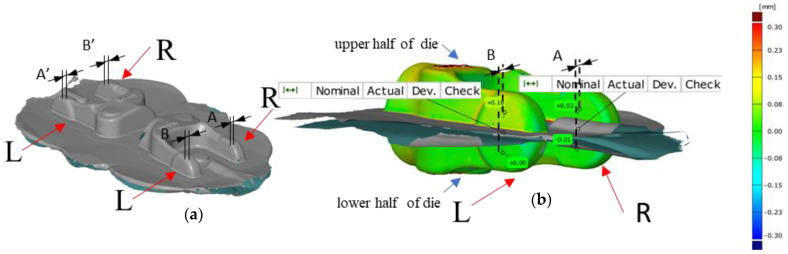
Exemplary results of the joggle defect analysis: (**a**) the whole forging, (**b**) half of the forging, shown on a colour map of deviations.

Figure 16a shows the assumed denotations for the joggle analysis and an exemplary compilation of results for the joggle defect for the whole forging consisting of two yokes forged in a two-component system. By means of the denotations assumed in Figure 16b, exemplary results of the analysis of data obtained from 3D scanning for the joggle defect of a single forging from a two-component system were demonstrated (Figure 15).

Figure 16b shows the two arms of the yoke—left (L) and right (R). For the analysis, four points were selected (Figure 16b), two on each half (upper and lower) of the forging of the left (L) and right (R) arm, lying at a symmetric distance from the parting plane of the nominal forging. In the upper portion of the left arm, we can notice a deviation at the level + 0.18 mm, whereas in the lower one—0.00 mm, which globally causes the formation of the joggle defect (denoted as B) measured along the nominal axis X at the level of 0.18 mm. Analogically, on the right arm at the top, we can see a deviation at the level of plus 0.03 mm, and at the bottom, minus −0.01 mm, which causes the formation of the joggle defect (denoted as A) measured along the nominal axis X at the level of 0.02 mm. The difference in the results for the left arm L and right arm R is caused by an asymmetric shift of the upper half and the lower die, which proves the existence of joggling and twisting.

**Figure 17:**
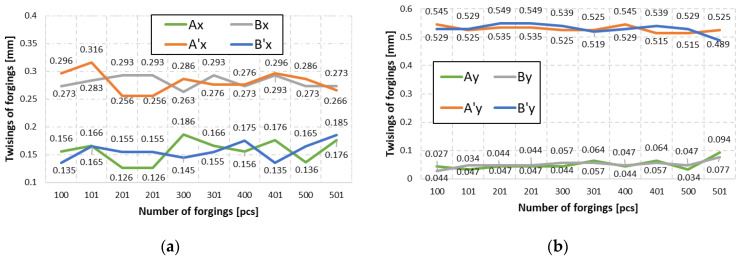
Diagram of the joggle values of two consecutive forgings cyclically collected every 100 items from the process, for the forging bay with geometrical defects: (**a**) components calculated in axis X, (**b**) components calculated in axis Y.

To determine the resultant of the position deviation occurring at the moment when the slide is located in the extreme lower position consisting of a joggle component X and Y, it is necessary to determine the joggle value in the direction of axis Y analogically to the way it was carried out for the direction X (Figure 17).

To that end, a 3D scanning measurement was performed for two consecutive forgings, which were cyclically collected every 100 items from the process, from the instrumentation (dimensions before correction) with geometrical defects in the form of joggle. Based on the analysis of the measurement data, diagrams were developed for forging series I (Figure 17) of the values describing the components X and Y of the joggle for four points A, B A’ and B’ of two consecutive forgings cyclically collected every 100 items from the process, from the instrumentation with geometrical defects.

**Figure 18:**
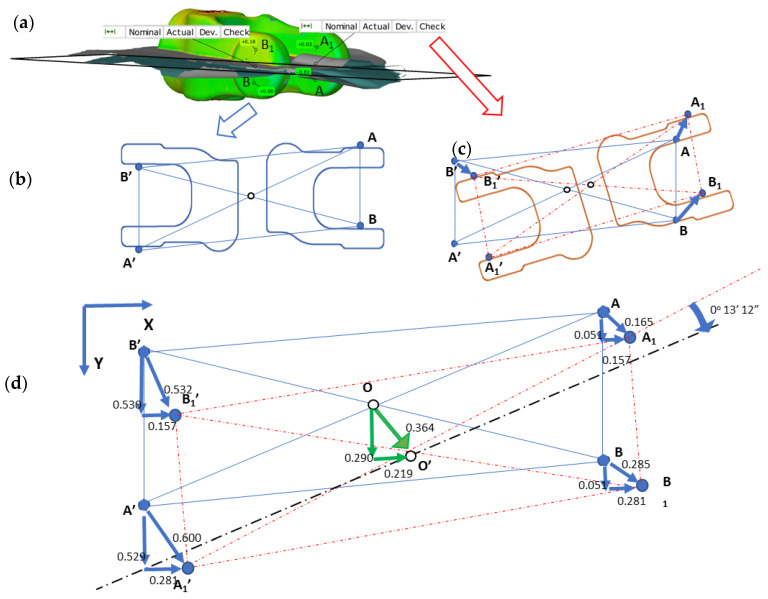
(**a**) The results of the analysis of the joggle defect for a single yoke, a diagram of a forging from a two-component system: (**b**) lower half of the die with the nominal position of the points, (**c**) lower half of the die with resultant joggle vectors, (**d**) diagram of dimensional compensation with calculated values of mean joggle necessary to determine the corrections.

**Figure 19:**
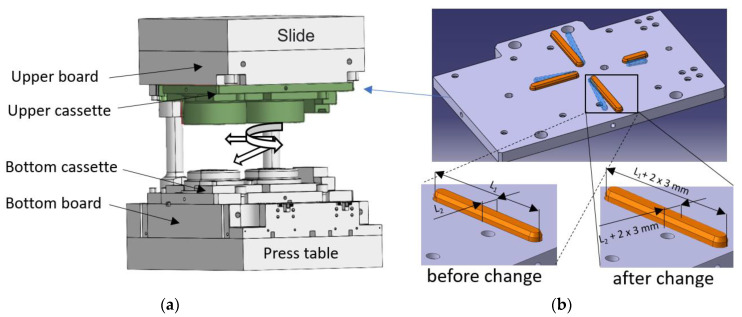
Concept of geometrical changes shown on a schematic diagram of the upper cassette SMED system: (**a**) the working space of the press, (**b**) introduced changes (the original arrangement/orientation of keyways are schematically marked in blue).

For each of the four pairs of values calculated for two consecutive forgings (e.g., the 100th and 101st) cyclically collected every 100 items, we can notice that, for example, the shift of point B in both axes, X and Y, is larger than the value of acceptable deviation for the forging (0.2 mm). Moreover, based on other measurement points, we can see that their deviations in both directions are different, which suggests the presence of joggle and the twisting of the upper portion of the forging in respect of the lower one. The good news acquired from the performed analysis is that the particular values change, yet their scatter is not higher than 0.04 mm, which additionally proves the repeatability of the process. In order to increase the accuracy and eliminate the errors, a decision was made to average the values for each of the analyzed points separately and assume the average value of the joggle vectors for further analysis. This way, the joggle resultants were obtained for four selected points (blue vectors), shown in Figure 18c, in respect of the nominal positions of the points of the lower half of the forging (die) (Figure 18b).The obtained results, after being connected with lines, make it possible to construct a characteristic geometry in the form of a rhombus ABA’B’ (Figure 18d). Additionally, on the intersection of lines A’–D’ and B’–C’, point O’ was marked for the upper half of the die and analogically for the nominal positions O of the lower half of the die. Also, to correct the tool geometry, it is necessary to relocate the rhombus constructed based on the values described by the joggle parameterA_1_B_1_A_1′_B_1′_ (Figure 18c, upper half of the forging) to the position created based on the nominal values (Figure 17b, lower half of the forging in the form of a rhombus ABA’B’). After the rhombi have been relocated, the center O will overlap with O’, which will enable a rotation by the angle ∝ marked in the figure (Figure 18d). This made it possible to determine the two desired values of the joggle vector components OO’x = 0.219 mm and OO’y = 0.290 mm. Additionally, it was established that the twist axis crosses point O’ by the angle ∝ = 0.3145°. The analysis was performed with the use of CAD systems (solid_works).The determined values describe the shift together with the twist of the upper and lower half of the forging, which have been additionally shown in Figure 19a as well as the concept of introducing changes in geometry. To that end, a decision was made to perform a correction of the wedges and spline ways (Figure 19b) connecting the upper and lower SMED board causing a shift according to the values of the vector components OO’x = 0.219 mm and OO’y= 0.290 mm, as well as a rotation around the axis crossing the point of the angle ∝ = 0.3145° (Figure 18a).

The changes consisted of widening the spline ways by as much as 3 mm per side as well as increasing their length (considering the new orientation) in order to increase the rigidity and stability of the connection between the cases and the boards. Introducing those changes makes it possible to correct the position and orientation of the upper and lower portion of the tools in respect of each other (Figure 19).

Such changes were introduced as the upper board and the slide were deemed as “large” and rigid elements of big masses, which causes their increased rigidity in respect of smaller boards of the SMED instrument. Additionally, the connection between the upper and lower board is beyond the guide posts, which increase the rigidity of the construction.

**Figure 20:**
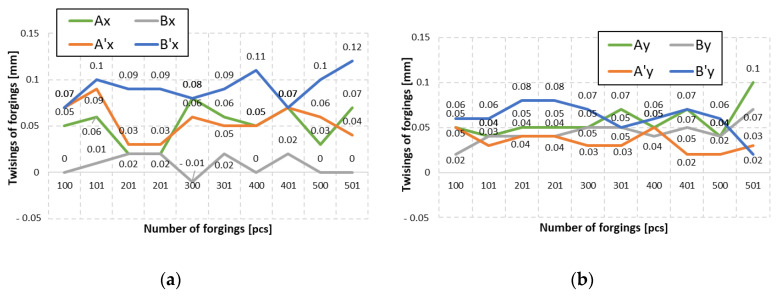
Diagram of the joggle values of two consecutive forgings cyclically collected every 100 items from the process, for a forging bay with corrected geometry: (**a**) components calculated in axis X, (**b**) components calculated in axis Y.

**Figure 21:**
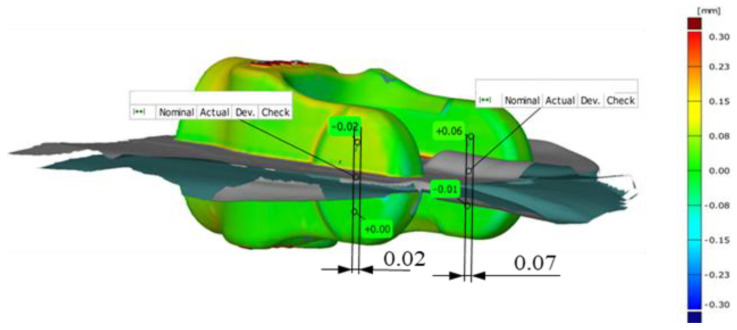
Exemplary results of a colour map of deviations with marked reduced joggle and twist values as a result of correctional changes in the kinematic chain.

To verify the correctness of the introduced tool changes, another analysis of measurement data was performed for series II of forgings made by means of the tools with reduced geometry. The performed measurements with the use of the 3D scanning method made it possible to obtain results in the form of diagrams for the joggle value of two consecutive forgings cyclically collected every 100 items from the process, for the forging bay with a corrected geometry of the components X and Y (Figure 20). The obtained values confirm that the introduced changes make it possible to significantly reduce the errors by 65% in respect of the joggle values before their introduction. Figure 21 shows exemplary results of a colour map of deviations with marked reduced joggle values determined from collected forgings in second series (Figure 15f–j).

The presented results confirm that introducing the proposed geometrical changes into the kinematic chain, determined based on the performed investigations by means of an indirect method consisting in measuring and analyzing the geometry of cyclically collected forgings, make it possible to eliminate the forging defects. It should be noted that changes in the connections were introduced only on the upper side of the kinematic chain, as, based on the obtained results (Figure 20), this ensured the forgings staying within the tolerance field equaling 0.2 mm with “large margins”. It may be that introducing a similar solution into the lower portion of the kinematic chain would cause even more advantageous changes; however, there was a concern of the side forces present during the forging process making the system over rigid as a result of long forging series and thus causing accelerated wear of the spline ways and wedges. This, however, is going to be the subject of further studies.

A novelty of the presented method in contrast to different approaches base of modern, and expensive, advanced measurement methods, is to use the results of the scanning of forgings (indirect method) as an effective solution to the problem of forgings defects, which could not be solved in a classic way using many advanced methods, including: dynamic system of deformation analysis, numerical modeling and as well as direct 3D scanning of key elements of the press and forging equipment.

## 4. Conclusions

The article proposes a proprietary indirect method consisting in determining difficult to eliminate forging defects in the form of joggles and twists of yoke-type forgings in an automated two-component system on a crank press. As it has been demonstrated, the results of the performed studies with the use of 3D scanning techniques of key elements of the press, which may have caused forging defects, did not demonstrate any difference in respect of the technical documentation and were within the assumed tolerance fields. Nevertheless, both the investigations, with the use of numerical modelling of the hot die forging process itself and those performed by means of a dynamic displacement measurement system showed that, in the case of this type of long yoked forgings, relatively small (in respect of the main direction) horizontal side forces occur, which, with very fast deformation (0.2 s) and high inertia of the whole system (many large rotating masses), can be of significant importance.

The results obtained from the system for a dynamic displacement analysis show that, at short time distances, as a result of the movement of the upper tools in respect of the lower ones, and with the assumption of the reference base both on the upper and lower tools, the top portion of the press shifts much more than its bottom portion. The highest shifts take place during the roughing operation (middle operation), which was also confirmed by the results of numerical modelling. The maximal displacement values of the tools reached even over 0.3 mm in the horizontal plane. Higher displacement values were observed during the loading, that is right after deformation, which could have been caused by seizure of the forgings as a result of their complicated shape. Despite the numerous performed tests and their analyses based on the classic approach to the analysis of a complicated issue, due to a very complex kinematic chain, it was unfortunately impossible to perform a thorough diagnostic of the problem of forging defects occurring on the modernized forging bay.

Herein, the authors have proposed a method of indirect measurements, consisting of a dimensional and shape analysis of the results of measurements obtained from 3D scanning for a few variants of production cycles, which make it possible to obtain the information necessary to correct the geometry of the kinematic chain. The performed analysis enabled the determination of the component values of the displacement vector of two halves of the die in respect of each other OO’x = 0.219 mm and OO’y = 0.290 mm as well as the torsional angle ∝ = 0.3145° (Figure 18). To perform a correction of the kinematic chain’s geometry, a decision was made to introduce changes into the geometry of the wedges and splineways (Figure 19b), which connect the upper and lower board with the upper SMED board, causing the planned displacement of the die halves in respect of each other. The introduced changes consisted in widening the splineways by as much as 3 mm per side, as well as increasing their length (considering the new orientation) to increase the rigidity and stability of the connection between the cases and the boards. Introducing such changes makes it possible to correct the position and orientation of the upper and lower portion of the tools in respect of each other. Additionally, the shapes of the wedges were changed from the conic ones to wider flatter ones (with a 30-degree phase on the height and next with a flat surface), which during the designing assumptions, should facilitate the assembly of the SMED tool in the upper board, but could also introduce additional yet small displacements and torsions of the forgings. The obtaining of geometrically and qualitatively correct products was verified by an analysis of another identical series of forgings.

The future directions of research will be to verify the proposed approach and use the developed method to analyze similar problems for the processes of forging other forgings. Further development and application of 3D scanning to other industrial applications is also planned, because, as shown in the state of the art (especially in the authors’ works), measurements using spatial scanning methods have great research potential.

## Figures and Tables

**Table 1:** Chemical compositions for tool steel and workpieces.

Material	C	Mn	Si	P	S	Cr	Ni	Mo	W	V	Cu	Fe
C45	0.42–0.5	0.5–0.8	0.1–0.4	max 0.04	max 0.04	max 0.3	max 0.3	max 0.1	-	-	max 0.3	rest

## Data Availability

Data sharing not applicable.
